# Knowledge of dentists, dental auxiliaries, and students regarding the COVID-19 pandemic in Saudi Arabia: a cross-sectional survey

**DOI:** 10.1186/s12903-020-01361-7

**Published:** 2020-12-21

**Authors:** Suliman Y. Shahin, Amr S. Bugshan, Khalid S. Almulhim, Mishali S. AlSharief, Yousif A. Al-Dulaijan, Intisar Siddiqui, Faisal D. al-Qarni

**Affiliations:** 1 Department of Preventive Dental Sciences, College of Dentistry, Imam Abdulrahman Bin Faisal University, Dammam, Saudi Arabia; 2 Department of Biomedical Dental Sciences, College of Dentistry, Imam Abdulrahman Bin Faisal University, Dammam, Saudi Arabia; 3 Department of Restorative Dental Sciences, College of Dentistry, Imam Abdulrahman Bin Faisal University, Dammam, Saudi Arabia; 4 Department of Substitutive Dental Sciences, College of Dentistry, Imam Abdulrahman Bin Faisal University, Dammam, Saudi Arabia; 5 Vice Deanship for Studies, Development and Community Service, College of Dentistry, Imam Abdulrahman Bin Faisal University, Dammam, Saudi Arabia

**Keywords:** Awareness, Dentists, Dental student, COVID-19, SARS-CoV-2

## Abstract

**Background:**

This study aimed to assess the knowledge of dental professionals in Saudi Arabia regarding severe acute respiratory syndrome coronavirus 2 (SARS-CoV-2) and coronavirus disease 2019 (COVID-19).

**Methods:**

A questionnaire was developed to assess various dental professionals from both governmental and private sectors through online and social media outlets.

**Results:**

A total of 1,033 questionnaires were collected (273 dental students, 193 dental auxiliary personnel, 544 dentists). In all, 63.4% of the respondents worked in hospitals. Of all the respondents, 44.9%, 33.4%, and 21.7% worked in governmental clinics, academia, and the private sector, respectively. Overall knowledge of the incubation period and route of transmission of SARS-CoV-2 was consistent across all dental professions. Knowledge of hand-soap cleaning time was significantly different among dental professionals (*p* < 0.001). Dental professionals displayed significant disagreement on the survival of SARS-CoV-2 outside the host (*p* < 0.001). Furthermore, 75.1% of the respondents were reluctant to treat a suspected COVID-19 patient, and 92% of the participants believed that the mode of transmission was droplet inhalation. Fever, coughing, and shortness of breath were identified as the most common symptoms of COVID-19. Most standard methods of prevention in the dental office were selected by at least 50% of the participants.

**Conclusions:**

Dental professionals seem to be consistent regarding their knowledge of the incubation period of SARS-CoV-2. However, knowledge of viral survivability and recommended hand-soap washing time was significantly variable among the professionals. A high degree of apprehension toward suspected COVID-19 patients existed among all dental professionals. Pandemic-awareness campaigns are essential among healthcare providers.

## Background

On January 30, 2020, the World Health Organization (WHO) [1] announced a public health emergency of international significance regarding a global pneumonia epidemic. The WHO called this disease the novel viral pneumonia coronavirus disease 2019 (COVID-19) on February 11, 2020. The first few cases of COVID-19 were identified in December 2019 in Wuhan, China, after genome sequencing of samples from patients presenting with pneumonia symptoms of unknown etiology [1]. The virus then spread to other provinces of China within weeks and became a global pandemic within a few months [2]. On March 2, 2020, Saudi Arabia announced its first case of severe acute respiratory syndrome coronavirus 2 (SARS-CoV-2) infection. Since then, the Saudi Ministry of Health has urged the general population to engage in social distancing and adopt sanitary habits and self-quarantine for a period of 14 days if an individual has recently been residing out of the kingdom. Coronaviruses are enveloped positive-strand RNA viruses with a crown-like appearance due to the presence of glycoprotein projections on their surfaces [3]. SARS-CoV-2 shares the same host receptor with SARS-CoV, namely, human angiotensin-converting enzyme 2. The natural host of SARS-CoV-2 may be the bat, as it showed 96.2% whole-genome identity to BatCoV RaTG13 [4].

The common clinical symptoms of patients with novel viral pneumonia include fever, cough, myalgia, and exhaustion with an irregular chest computed tomography scan; less common symptoms are sputum development, headache, hemoptysis, and diarrhea [5, 6]. The typical transmission routes of novel coronaviruses include direct (coughing, sneezing, and droplet inhalation) and contact (with oral, nasal, and eye mucous membranes) transmission [7].

A dental professional team is considered a high-risk group. Pathogenic microorganisms may be transmitted in dental settings through inhalation of airborne microorganisms that can stay trapped in the air for long periods [8]; direct contact with blood, oral fluids, or other patient materials [9]; contact of the conjunctival, nasal, or oral mucosa with droplets and microorganism-containing aerosols produced from an infected person and propelled by coughing and talking at a short distance without a mask [10, 11] or using high-pressure irrigation systems such as the handpiece or ultrasonic scalers; and indirect contact with contaminated instruments and/or environmental surfaces [12]. Therefore, it is crucial for dental professional teams to practice preventive measures against SARS-CoV-2 infection by focusing on hand hygiene, with an adequate hand-soap cleaning time of approximately 40 s [13], and personal protective equipment (PPE); ensuring an adequately ventilated space; and exercising caution when performing aerosol-generating procedures [11].

The aim of this study was to assess the knowledge of dentists and dental auxiliaries and students in Saudi Arabia regarding SARS-CoV-2, as they are considered a high-risk group for infection, and investigate the potential gaps in knowledge that may affect health and safety in the dental workplace.

## Methods

A cross-sectional survey was conducted after ethical approval (EA# 202049) was obtained from the Scientific Research Unit at the College of Dentistry, Imam Abdulrahman Bin Faisal University, Dammam, Saudi Arabia. The questionnaire was developed in English and further reviewed for face validation by two independent local reviewers. Major governmental hospitals, private clinics, and academic universities in various cities of all provinces of Saudi Arabia were approached. A corresponding link was sent to all targeted samples using different methods, including emails, text messages, and social media outlets. The targeted samples included dental students, dental interns, dental hygienists, dental assistants, general dentists, dental specialists, and consultants working in hospital and nonhospital governmental and private sectors. Respondents who were from outside Saudi Arabia and those who missed more than two questions were excluded from the study. To ensure the clarity and validity of the questions, a pilot test of the questionnaire was initially performed on a group of 100 dental professionals. The questionnaire was attached as Additional file 1.

The survey was constructed based on the latest literature resources about the characteristics and routes of transmission of SARS-CoV-2 and the signs and symptoms and prevention methods of COVID-19 [1–9, 13–16]. The first part of the questionnaire included the demographic information of the participants, that is, their age group, sex, geographic location of practice within Saudi Arabia, type of profession, primary work sector (academia, government, or private), and work setting (hospital or nonhospital). The second part of the questionnaire assessed the participants’ awareness of the basic knowledge of SARS-CoV-2 and COVID-19. Dental professionals were asked about COVID-19 signs and symptoms and SARS-CoV-2 routes of transmission, incubation period, and viability outside the human body. The last part of the questionnaire included the methods of prevention and their attitude toward treating SARS-CoV-2-infected patients. All data were collected during March 2020.

SPSS-20.0 was used for statistical data analysis (IBM Corp. Released 2011. IBM SPSS Statistics for Macintosh, Version 20.0. Armonk, NY, USA: IBM Corp.). The results of all categorical variables are presented in terms of frequencies and percentages. The chi-square test was performed to compare the factors between work sectors, work settings, and professionals. All tests were performed at a significance level of α = 0.05.

## Results

Out of 1033 questionnaires received, 20 were omitted because they were from outside Saudi Arabia. Some of the questionnaires were incomplete; those with more than one missing answer were excluded from the analysis.

### Reliability and validity

A pilot sample size of 100 participants was selected for the reliability and validity of the research instrument. After standardizing all items, reasonably good interrater reliability was found (Cronbach’s α  = 0.788). Face validation of the questionnaire was performed by two independent local experts.

### Demographic results

More than half of the participants were female, accounting for 53.5% (n = 542) of the study sample, while men accounted for 46.5% (n = 471) of the study sample. Half of the participants were 20 to < 30 years old (50%), 301 were 30 to < 40 years old (29.7%), 134 were 40 to < 50 years old (13.2%), and 71 were ≥ 50 years old (7%). Among the professions, the highest number of respondents were specialists/consultants (n = 271, 26.8%), followed by dental students (n = 197, 19.5%), general dentists (n = 181, 17.9%), dental assistants (n = 156, 15.4%), postgraduate residents (n = 92, 9.1%), dental interns (n = 76, 7.5%), and dental hygienists (n = 37, 3.7%). Detailed distribution of participants’ demographic characteristics is listed in Table 1.

**Table 1:** Distribution of demographic characteristics of study participants

Variable	n (%)
*Age (years)*	
20 to < 30	507 (50%)
30 to < 40	301 (29.7%)
40 to < 50	134 (13.2%)
50 or above	71 (7%)
*Profession*	
Dental student	197 (19.5%)
Dental intern	76 (7.5%)
Dental assistant	156 (15.4%)
Dental hygienist	37 (3.7%)
Postgraduate resident	92 (9.1%)
General dentist	181 (17.9%)
Specialist/consultant	271 (26.8%)
*Region*	
Northern Region	46 (4.5%)
Southern Region	107 (10.6%)
Western Region	226 (22.3%)
Eastern Region	367 (36.2%)
Central Region	267 (26.4%)
*Work sector*	
Academia	336 (33.4%)
Government	452 (44.9%)
Private	219 (21.7%)
*Work setting*	
Hospital	641 (63.4%)
Nonhospital	370 (36.6%)

### Awareness of SARS-CoV-2 and bivariate analysis results

When evaluating the awareness of the survival period of SARS-CoV-2 outside a host, approximately half of the respondents (n = 496, 49.2%) believed that the survivability of the virus outside the host was a couple of hours, 360 (35.7%) believed that the survivability of the virus was a couple of days, 71 (7%) believed it was a couple of weeks, and 82 (8.1%) did not know. In the between-group comparison, knowledge of the survivability of SARS-CoV-2 outside a host among those working in different settings and sectors was not significantly different (*p* > 0.05). However, when evaluating the various dental professions, a statistically significant difference was found among the various professions (*p* < 0.001). Among dental assistants, 17.9% (n = 28) believed that the survivability of SARS-CoV-2 was a couple of weeks, whereas only between 2.8% and 7.6% of the rest of the dental professionals believed the same concept. Furthermore, 48.3% (n = 131) of specialists and consultants believed that the survivability of SARS-CoV-2 was a couple of days, while only 23.7% (n = 18), 25.6% (n = 40), and 26.4% (n = 52) of the interns, assistants, and students believed the same concept. This indicates a statistically significant disagreement among dental professionals regarding the survivability of SARS-CoV-2 outside the host.

The awareness of the recommended hand-soap cleaning time to prevent SARS-CoV-2 infection was distributed as follows: 561 (55.6%) responded 40 s; 277 (27.4%), 20 s; 155 (15.3%), 60 s; and 17 (1.7%) responded that they did not know the recommended hand-soap cleaning time for prevention. Again, in the between-group comparison, knowledge of the recommended hand-soap cleaning time to prevent SARS-CoV-2 infection among those working in different settings and sectors was not significantly different (*p* > 0.05). However, when evaluating the various dental professions, a statistically significant difference was found among the various professions with regard to the recommended hand-soap cleaning time (*p* < 0.001). The majority of dental professionals, except for dental assistants, responded in the range of 55.9%–69.4% on a recommended time of 40 s, whereas only 25% (n = 39) of dental assistants answered the recommended 40 s of hand-soap cleaning time to prevent SARS-CoV-2 contamination.

Regarding the questions about willingness to treat a suspected COVID-19 patient, approximately three-quarters of dental professionals (n = 756, 75.1%) did not want to treat a suspected COVID-19 patient. Bivariate analyses for between-group comparisons among those working in different settings and sectors and among the various dental professions regarding this question were not significantly different (*p* > 0.05). Table 2 and Table 3 illustrate the knowledge and awareness of SARS-CoV-2 and COVID-19 by work setting and sector and by dental profession. The Pearson chi-square results are shown as well.

**Table 2:** Pearson chi-square results for the knowledge and awareness of SARS-CoV-2 and COVID-19 by profession

Variable	Dental profession								*p*
	Dental student	Dental intern	Dental assistant	Dental hygienist	Postgraduate resident	General dentist	Specialist consultant	Total	
*Q1: Incubation period of SARS-CoV-2?*									
1–7 days	3 (1.5%)	2 (2.6%)	4 (2.6%)	1 (2.8%)	4 (4.4%)	4 (2.2%)	9 (3.3%)	27	
1–14 days	184 (93.4%)	71 (93.4%)	139 (89.1%)	35 (97.2%)	84 (91.2%)	165 (91.7%)	245 (90.4%)	923	0.866
1–21 days	7 (3.6%)	3 (4.0%)	12 (7.7%)	0 (0%)	4 (4.4%)	10 (5.6%)	15 (5.6%	51	
Do not know	3 (1.5%)	0 (0%)	1 (0.6%)	0 (0%)	0 (0%)	1 (0.5%)	2 (0.7%)	7	
Total	197 (100%)	76 (100%)	156 (100%)	36 (100%)	92 (100%)	180 (100%)	271 (100%)	1008	
*Q2: Survival of SARS-CoV-2 outside the host?*									
Couple of hours	108 (54.8%)	48 (63.2%)	78 (50%)	22 (61.1%)	42 (45.6%)	83 (46.1%)	115 (42.4%)	496	
Couple of days	52 (26.4%)	18 (23.7%)	40 (25.6%)	11 (30.5%)	39 (42.4%)	69 (38.3%)	131 (48.3%)	360	0.000 **
Couple of weeks	10 (5.1%)	3 (3.9%)	28 (18%)	1 (2.8%)	7 (7.6%)	10 (0.6%)	12 (4.5%)	71	
Do not know	27 (13.7%)	7 (9.2%)	10 (6.4%)	2 (5.6%)	4 (4.4%)	18 (10%)	13 (4.8%)	81	
Total	197 (100%)	76 (100%)	156 (100%)	36 (100%)	92 (100%)	180 (100%)	271 (100%)	1008	
*Q3: Recommended hand-soap cleaning time to prevent SARS-CoV-2-infection?*									
Approximately 20 s	35 (17.8%)	15 (19.7%)	60 (38.5%)	5 (13.9%)	19 (20.7%)	45 (24.8%)	98 (36.3%)	277	
Approximately 40 s	118 (60.3%)	44 (57.9%)	39 (25.0%)	25 (69.4%)	63 (68.4%)	121 (66.9%)	151 (55.9%)	561	
Approximately 60 s	38 (19.3%)	16 (21.1%)	56 (35.9%)	4 (11.1%)	6 (6.5%)	14 (7.7%)	18 (6.7%)	152	0.000**
Do not know	5 (2.6%)	1 (1.3%)	1 (0.6%)	2 (5.6%)	4 (4.4%)	1 (0.6%)	3 (1.1%)	17	
Total	196 (100%)	76 (100%)	156 (100%)	36 (100%)	92 (100%)	181 (100%)	270 (100%)	1007	
*Q4: Willingness to treat a suspected COVID-19 patient?*									
Yes	52 (26.4%)	15 (19.7%)	44 (28.8%)	14 (38.9%)	16 (17.4%)	48 (26.7%)	62 (23%)	251	
No	145 (73.6%)	61 (80.3%)	109 (71.2%)	22 (61.1%)	76 (82.6%)	132 (73.3%)	208 (77%)	753	0.129
Total	197 (100%)	76 (100%)	153 (100%)	36 (100%)	92 (100%)	180 (100%)	270 (100%)	1004	

All tests were performed at a significance level of α = 0.05

^*^Significant at *p* < 0.05

^**^Significant at *p* < 0.001

**Table 3:** Pearson chi-square results for the knowledge and awareness of SARS-CoV-2 and COVID-19 by work settings and sectors

Variable	Work setting			*p*	Work sector				*p*
	Hospital	Nonhospital	Total		Academia	Government	Private	Total	
*Q1: Incubation period of SARS-CoV-2?*									
1–7 days	18 (2.8%)	9 (2.4%)	27		8 (2.4%)	10 (2.2%)	9 (4.1%)	27	
1–14 days	582 (91.0%)	342 (92.7%)	924	0.587	309 (92.0%)	418 (92.7%)	193 (88.5%)	920	0.648
1–21 days	34 (5.3%)	17(4.6%)	51		16 (4.7%)	21 (4.7%)	14 (6.4%)	51	
Do not know	6 (0.9%)	1(0.3%)	7		3 (0.9%)	2 (0.4%)	2 (1.0%)	7	
Total	640 (100%)	369 (100%)	1009		336 (100%)	451 (100%)	218 (100%)	1005	
*Q2: Survival of SARS-CoV-2 outside the host?*									
Couple of hours	323 (50.5%)	173 (46.9%)	496	0.146	163 (48.5%)	222 (49.3%)	111 (50.7%)	496	
Couple of days	213 (33.3%)	147 (39.8%)	360		133 (39.6%)	157 (34.9%)	67 (30.6%)	357	0.083
Couple of weeks	46 (7.2%)	25 (6.8%)	71		15 (4.5%)	39 (8.7%)	17 (7.8%)	71	
Do not know	58 (9.0%)	24 (6.5%)	82		25 (7.4%)	32 (7.1%)	24 (10.9%)	81	
Total	640 (100%)	369 (100%)	1009		336 (100%)	450 (100%)	219 (100%)	1005	
*Q3: Recommended hand-soap cleaning time to prevent SARS-CoV-2 infection?*									
Approximately 20 s	163 (25.5%)	113 (30.5%)	276		95 (28.4%)	124 (27.5%)	56 (25.6%)	275	
Approximately 40 s	359 (56.2%)	201 (54.3%)	560	0.244	179 (53.6%)	251 (55.7%)	128 (58.4%)	558	0.060
Approximately 60 s	106 (16.6%)	49 (13.2%)	155		48 (14.4%)	73 (16.2%)	33 (15.1%)	154	
Do not know	10 (2.7%)	7 (2.0%)	17		12 (3.6%)	3 (0.6%)	2 (0.9%)	17	
Total	638 (100%)	370 (100%)	1008		334 (100%)	451 (100%)	219 (100%)	1004	
*Q4: Willingness to treat a suspected COVID-19 patient?*									
Yes	164 (25.7%)	87 (23.7%)	251	0.489	90 (26.9%)	113 (25.1%)	47 (21.6%)	250	
No	475 (74.3%)	280 (76.3%)	755		244 (73.1%)	337 (74.9%)	171 (78.4%)	752	0.358
Total	639 (100%)	367 (100%)	1006		334 (100%)	450 (100%)	218 (100%)	1002	

The majority of the 1,013 participants (n = 932, 92%) believed that the route of transmission was through droplet spread (sneezing or coughing). Approximately half of the participants (n = 548 dental professionals, 54.1%) believed that the virus could be transmitted through direct contact with saliva, blood, or other body fluids. Moreover, only approximately one-third of the respondents believed that transmission could occur via direct skin-to-skin contact and airborne transmission, with 312 (30.8%) and 305 (30.1%) responses, respectively.

Almost all of the respondents believed fever to be a sign and symptom of COVID-19, with 1,000 responses (98.7%). Furthermore, 944 (93.2%) respondents believed coughing to be a sign and symptom. In addition, 940 (92.8%) respondents believed shortness of breath was a common sign and symptom of the disease. The responses for the remaining COVID-19 signs and symptoms were as follows: 736 (72.7%) responses for headache; 671 (66.2%), shortness of breath; 492 (48.6%), muscle pain; 323 (31.9%), diarrhea; 295 (29.1%), nasal congestion; 187 (18.5%), vomiting; and 32 (3.2%), skin rash.

The methods of SARS-CoV-2 transmission prevention in dental clinics among participants were believed to be as follows (from the highest response to the lowest): PPE (gloves, masks, and wrapping) with 973 (96.1%) responses; hand-soap cleaning, 960 (94.8%); clinic surface disinfection, 877 (86.6%); hand sanitizers, 873 (86.2%); rubber dam isolation, 620 (61.2%); utilization of an isolated clinic, 592 (58.4%); adequate ventilation, 579 (57.2%); use of preoperative chlorhexidine mouthwash, 360 (35.5%); and use of preoperative hydrogen peroxide mouthwash, 207 (20.4%). Table 4 summarizes the responses of all dental professionals with regard to awareness of the route of SARS-CoV-2 transmission, signs and symptoms of COVID-19, and methods of transmission prevention in dental clinics.

**Table 4:** A summary of the awareness of the route of SARS-CoV-2 transmission, signs and symptoms of COVID-19, and methods of SARS-CoV-2 transmission prevention in dental clinics

	Frequency	Percent
*Q5: Route of SARS-CoV-2 transmission*		
Direct skin-to-skin transmission	312	30.8
Direct fluid transmission (saliva, body fluids, or blood)	548	54.1
Droplet spread (sneezing or coughing)	932	92
Airborne transmission (air or dust)	305	30.1
*Q6: Signs and symptoms of COVID-19*		
Fever	1000	98.7
Headache	736	72.7
Diarrhea	323	31.9
Vomiting	187	18.5
Muscle pain	492	48.6
Coughing	944	93.2
Shortness of breath	940	92.8
Sore throat	671	66.2
Nasal congestion	295	29.1
Skin rash	32	3.2
*Q7: Methods of SARS-CoV-2 transmission prevention in dental clinics*		
Hand-soap cleaning	960	94.8
Hand sanitizers	873	86.2
Personal protective equipment (gloves, masks, and wrapping)	973	96.1
Preoperative chlorhexidine mouthwash	360	35.5
Preoperative hydrogen peroxide mouthwash	207	20.4
Rubber dam isolation	620	61.2
Clinic surface disinfection	877	86.6
Adequate ventilation	579	57.2
Utilization of an isolated clinic	592	58.4

Logistic regression analysis was performed by taking the willingness of the dentist to treat suspected COVID-19 patients as an independent binary variable and sex, age, practice status, work sector, and dental service set up as independent covariates to evaluate the effect of these covariates on the outcome variable related to the perception of dental professionals regarding treating suspected COVID-19 patients.

The analysis revealed 2 predictors of willingness to treat suspected COVID-19 patients, i.e., dental assistants (*p* = 0.047) and dental hygienists (*p* = 0.012). Postgraduate residents were the least willing to treat COVID-19-suspected patients, whereas dental assistants were 2 times (OR = 1.92) and dental hygienists were 3 times (OR = 3.02) more likely willing than postgraduate residents to treat COVID-19-suspected patients. There was a nonsignificant association of willingness of dentists to treat COVID-19 patients with regards to sex (OR = 1.12), younger versus older age (OR = 1.07), academic sector (OR = 1.34) and government sector (OR = 1.22) versus private sector, and hospital versus nonhospital setup (OR = 1.11). Table 5 highlights the odds ratio of willingness to treat suspected COVID-19 patients by various variables.

**Table 5:** Predictors of willingness of dentist to treat suspected Covid-19 patient

Factors	Willingness of dentist		Odd ratio	*p* value
	Yes (n = 251)	No (n = 756)	OR (95% CI)	Sig
Gender (Female)	129 (51.4)	409 (54.1)	1.12 (0.84–1.48)	0.456
Age (20 to < 30 years)	130 (51.8)	373 (49.3)	1.07 (0.73–1.55)	0.738
Age (30 to < 40 years)	71 (28.3)	230 (30.4)	0.94 (0.62–1.43)	0.788
Age (40 to < 50 years)	34 (13.5)	98 (13.0)	1.19 (0.60–2.35)	0.612
Age (> 50 years)	16 (6.4)	55 (7.3)	–	–
Dental student	52 (20.7)	145 (19.3)	1.70 (0.91–3.18)	0.095
Dental intern	15 (6.0)	61 (8.1)	1.17 (0.53–2.55)	0.697
Dental assistant	44 (17.5)*	109 (14.5)	1.92 (1.01–3.65)	0.047*
Dental hygienist	14 (17.5)*	22 (2.9)	3.02 (1.28–7.14)	0.012*
General dentist	48 (19.1)	132 (17.5)	1.73 (0.92–3.25)	0.090
Specialist/consultant	62 (24.7)	208 (27.6)	1.42 (0.77–2.60)	0.263
Postgraduate resident	16 (6.4)	76 (10.1)	–	–
Academic sector	90 (36.0)	244 (32.4)	1.34 (0.90–2.01)	0.153
Government sector	113 (45.2)	337 (44.8)	1.22 (0.83–1.80)	0.314
Hospital setup	164 (65.3)	475 (62.9)	1.11 (0.82–1.50)	0.489

*Indicates statistical significance

## Discussion

Due to its high mortality rate, SARS-CoV-2 and the associated debilitating COVID-19 are emerging topics that have recently received great attention and have undergone intense investigation. This pandemic disease is highly infectious, as the number of infected patients exceeded 2 million worldwide in < 6 months, as reported by the WHO [14].

This survey assessed the degree of awareness of dental professionals, as they are typically in close contact with patients when delivering oral healthcare services.

Based on our results, the overall knowledge about the incubation period, route of transmission, and recommended hand-soap cleaning time to prevent SARS-CoV-2 was consistent across all dental professions, except for dental assistants, where only 25% of them believed the recommended hand-soap cleaning time was 40 s. This clearly emphasizes the significance of the adequacy of viral prevention knowledge across all dental professionals and auxiliaries during pandemic periods. Overall, these results show an improvement in knowledge compared with the results found in a previous survey of dentists in Saudi Arabia about SARS-CoV [17]. Although modestly answered among all professionals, the highest response was among dental specialists/consultants, with only 48.3% believing that the survivability of SARS-CoV-2 outside the host was a couple of days. There seemed to be a significant disagreement among the dental professionals regarding the question of SARS-CoV-2 survivability (*p* < 0.001). Regarding general knowledge about SARS-CoV-2, our study showed a relatively lower percentage of individuals who answered “I do not know” to questions regarding the incubation period and recommended hand-soap cleaning time (0.69% and 1.69%, respectively) compared with viral survival outside the host (10.04%). This may be because local health authorities have utilized all media outlets toward a community-wide awareness campaign covering the pandemic, which had hand-soap cleaning time being one of the most shared preventive methods promoted. In contrast, the survival of the virus outside the host may be confusing due to various survival times related to different surfaces [8, 18]. This further emphasizes the significance of knowledge on viral survival to ensure adequate preventive measures in the working environment while treating suspected COVID-19 patients.

The majority of respondents, regardless of professional category, were reluctant to treat a patient under suspicion of having COVID-19, which indicated a certain level of apprehension. This is thought to be due to the general guidelines conveyed to caregivers by local authorities, that is, to limit dental procedures to urgent or emergency cases only and refrain from elective procedures. Regarding willingness to treat suspected patients with COVID-19, dental hygienists (38.89%) were most willing to treat suspected patients. In contrast, compared with the remaining groups, postgraduate dental residents (17.39%) were least willing. This is an interesting outcome, as dental hygiene is considered to have mostly elective procedures compared with general or specialized dentistry. Future studies addressing the level of apprehension among dental professionals are warranted.

Regarding the question about modes of transmission, the majority of the participants (92%) believed that the mode of transmission was droplet infection, followed by direct fluid transmission (54.1%) and direct skin-to-skin contact (30.8%). However, 30.1% of the participants also believed that the virus was airborne transmissible, which is clearly a misconception, as the disease is transmitted by aerosols [3]. The present study showed that the majority of the participants identified fever, coughing, and shortness of breath as the most common symptoms related to COVID-19 (98.7%, 93.2%, and 92.8%, respectively), which indicated a high degree of knowledge pertinent to this area. This is an intuitive finding considering that fever screenings have been increasingly prevalent at establishment entrances throughout the pandemic. Furthermore, previous epidemics of SARS-CoV and Middle East respiratory syndrome-related coronavirus (MERS-CoV) may explain the degree of knowledge regarding common respiratory symptoms of coronaviruses.

To prevent SARS-CoV-2 infection in dental clinics, dental professionals should follow standard precautions, which include appropriate hand hygiene, personal protective barriers (gloves, masks, face shields), use of preoperative mouthwash (1% hydrogen peroxide), rubber dam isolation, and implementation of effective and strict disinfection measures [15]. It is worth highlighting that 54.6% and 51.6% of dental assistants and hygienists, respectively, believed that the use of preoperative chlorhexidine mouthwash (0.12%), which is antibacterial, was a preventative measure against SARS-CoV-2. On the other hand, 84.9% and 93.5% of dental assistants and hygienists, respectively, did not believe that rubber dam isolation was a significant measure of SARS-CoV-2 prevention. This implies the presence of a gap in knowledge regarding preventive measures among the dental team.

Interestingly, dental assistants (*p* = 0.047) and dental hygienists (*p* = 0.012) had higher odds of willingness to treat suspected COVID-19 patients than postgraduate residents, who had the lowest willingness to treat these patients. This could be explained by less experience in dental practice in regard to residents in treating patients with medical conditions.

The representative sample of 1013 dental professionals covering all regions of Saudi Arabia is a strength of this study, as it is consistent with the distribution of the population within each region [16]. Furthermore, a thorough distribution among all dental professionals and dental work settings was achieved to act as a realistic representation of the country’s dental work force. To the best of our knowledge, this is the first representative survey to provide insight into the knowledge and perception of SARS-CoV-2 by dentists and dental auxiliaries and students in Saudi Arabia. Furthermore, the distribution of male and female participants was balanced (46.5% and 53.5%, respectively). However, this study has several limitations. In addition to the general limitations of a cross-sectional study and online surveys, one of our limitations was the dichotomous nature of the question as to whether the practitioner was willing to treat a SARS-CoV-2 patient. Information pertaining to participants who answered “No” but who would otherwise be willing to treat emergency patients only may have been lost, as the options were only “Yes” or “No,” especially with a considerable number of dental procedures being elective or at least of a nonurgent nature. In addition, with the high volume of information influx from media outlets in combination with a higher than average viewership due to curfews and lockdowns, the status quo of general perceptions may shift on a weekly or even daily basis, rendering this survey limited to a specific snapshot of time. A further limitation was the variation in the number of responses among the various dental professions. In addition, factors such as length of practice, nationality, and race were not considered in this study. Detailed investigation on practice in dental clinics in regard to the COVID-19 pandemic is warranted. Further studies addressing the abovementioned limitations are needed. This study can be used as a benchmark for general knowledge and attitudes toward pandemics in developing countries with an established dental healthcare system.

## Conclusion

The knowledge among dental professionals on SARS-CoV-2 and COVID-19 seems to be consistent when evaluating the viral incubation period. However, viral survivability and recommended hand-soap washing time were significantly variable among the professionals. Furthermore, a certain level of apprehension toward suspected COVID-19 patients existed among all dental professionals. Dental assistants and hygienists had higher odds of willingness to treat suspected COVID-19 patients. During a novel pandemic, a joint effort to strengthen knowledge among dentists and dental auxiliaries needs to be reinforced within the dental professional team. Studies comparing knowledge at every stage of the pandemic are recommended.

## Supplementary information

**Additional file 1**. The questionnaire distributed to the respondents.

## Data Availability

The datasets generated and analyzed during the current research are not publicly available, but they are available from the corresponding author on reasonable request.

## Abbreviations

SARS-CoV-2: Severe acute respiratory syndrome coronavirus 2
COVID-19: Coronavirus disease 2019
WHO: World Health Organization
PPE: Personal protective equipment
