# Supplementary material for: Knowledge of dentists, dental auxiliaries, and students regarding the COVID-19 pandemic in Saudi Arabia: a cross-sectional survey
Source: BMC Oral Health. 2020 Dec 21;20:363. doi: 10.1186/s12903-020-01361-7 (PMC7751262; doi:10.1186/s12903-020-01361-7)
Supplement: Supplementary file 1 — Additional file 1. The questionnaire distributed to the respondents. [file 12903_2020_1361_MOESM1_ESM.docx]

**Awareness of Dental Professionals in Saudi Arabia on COVID-19 Corona Virus**

This questionnaire is designed to measure awareness on COVID-19 among dental professionals in KSA.

It consists of 13 questions, and it will take around 5 minutes to answer all the questions.

Thank you for participating.

**Q1: Gender**

- Male
- Female

**Q2: Age group**

- Less than 20 years
- 20 to < 30 years
- 30 to < 40 years
- 40 to < 50 years
- 50 years and more

**Q3: Profession**

- Dental student
- Dental intern
- Dental assistant
- Dental hygienist
- Postgraduate resident
- General dentist
- Specialist/ Consultant

**Q4: Primary work sector**

- Academia
- Governmental
- Private

**Q5: Work setting?**

- Hospital
- Non-hospital

**Q6: Region of practice within Saudi Arabia**

- Northern region
- Southern region
- Western region
- Eastern region
- Central region
- Outside Saudi Arabia

**Q7: What is the incubation period of COVID-19?**

- 1-7 days
- 1-14 days
- 1-21 days
- I do not know

**Q8: Route of COVID-19 virus transmission (choose one or more)**

- Direct skin-skin transmission
- Direct fluid transmission (saliva, body fluids or blood)
- Droplet spread (sneezing or coughing)
- Airborne transmission (air or dust)

**Q9: How long does the COVID-19 virus survive outside the body?**

- Couple of hours
- Couple of days
- Couple of weeks
- I do not know

**Q10: Signs and symptoms of COVID-19 virus infection (choose one or more)**

- Fever
- Headache
- Diarrhea
- Vomiting
- Muscle pain
- Coughing
- Shortness of breath
- Sore throat
- Nasal congestion
- Skin rash

**Q11: Would you treat a suspected COVID-19 patient?**

- Yes
- No

**Q12: What are methods of COVID-19 transmission prevention in dental clinics (choose one or more)**

- Hand soap cleaning
- Hand sanitizers
- Personal protective equipment (gloves, masks and wrapping)
- Pre-operational chlorhexidine mouth rinse
- Pre-operational hydrogen peroxide mouth rinse
- Rubber dam isolation
- Clinic surface disinfection
- Adequate ventilation
- Isolated clinic

**Q13: How long is the recommended hand-soap cleaning time to prevent COVID-19 transmission?**

- About 20 seconds
- About 40 seconds
- About 60 seconds
- I do not know
